# Transoral Ultrasound in the Outpatient Clinic for the Diagnostic Work-Up of Oropharyngeal Cancer: A Feasibility Study

**DOI:** 10.3390/cancers15215292

**Published:** 2023-11-04

**Authors:** Martin Garset-Zamani, Rikke Norling, Christoffer Holst Hahn, Tina Klitmøller Agander, Christian von Buchwald, Tobias Todsen

**Affiliations:** 1 Department of Otorhinolaryngology, Head and Neck Surgery and Audiology, Copenhagen University Hospital—Rigshospitalet, 2100 Copenhagen, Denmark; 2 Institute of Clinical Medicine, Faculty of Health and Medical Sciences, University of Copenhagen, Blegdamsvej 3, 2200 Copenhagen, Denmark; 3 Department of Radiology, Copenhagen University Hospital—Rigshospitalet, 2100 Copenhagen, Denmark; 4 Department of Pathology, Copenhagen University Hospital—Rigshospitalet, 2100 Copenhagen, Denmark; 5 Copenhagen Academy for Medical Education and Simulation, The Capital Region of Denmark, 2100 Copenhagen, Denmark

**Keywords:** oropharyngeal cancer, head and neck cancer, squamous cell carcinoma, human papillomavirus, head and neck ultrasound, transoral ultrasound, intraoral ultrasound, transcervical ultrasound, neck ultrasound, oropharyngeal squamous cell carcinoma

## Abstract

**Simple Summary:**

Oropharyngeal cancers may be difficult to detect with current clinical and radiographic examinations. This study explores whether a new transoral ultrasound technique using high-resolution, small-footprint ultrasound transducers can improve the detection and staging of oropharyngeal cancers in an outpatient setting. We found that clinicians can use transoral ultrasound to improve the diagnostic workup of oropharyngeal cancers with an accuracy comparable to magnetic resonance imaging.

**Abstract:**

Magnetic resonance imaging (MRI) is the preferred imaging modality for oropharyngeal cancers (OPCs), but it has difficulties distinguishing between small OPCs and unilateral tonsil hypertrophy. We hypothesized that surgeon-performed transoral ultrasound (US) could be used to accurately detect T-stage OPCs. We performed a single-center prospective diagnostic accuracy study including patients with suspected or biopsy-verified OPCs during outpatient appointments. All patients were offered transoral US and MRI. If transoral US could not be tolerated by the patient, transcervical US was performed. The primary outcome was the diagnostic accuracy of detecting OPCs with US compared to MRI, using histopathology as the reference standard. The secondary outcome was comparing the primary tumor diameters between US and MRI blinded to each other. Out of the 26 patients included in the study, 21 (81%) had OPCs. Transoral US could be performed in 21/21 and 1/5 patients with suspected palatine and lingual tonsil OPCs, respectively. Overall, US diagnostic accuracy was 92%, compared to 81% with MRI (*p* = 0.37). US and MRI had a high correlation between tumor diameters in the anteroposterior diameter (R = 0.80, *p* < 0.001), corresponding to the depth axis on US. In conclusion, this small study showed the promise and feasibility of transoral US to improve the initial clinical evaluations of patients with suspected OPCs.

## 1. Introduction

Human papillomavirus (HPV) has caused a significant increase in the incidence of oropharyngeal cancers (OPCs) in recent years [1,2,3,4,5]. HPV-associated primary tumors are usually smaller and may be hidden below the surface of the palatine or lingual tonsils, where the clinical examination can be challenging [4,6,7]. Cross-sectional imaging, such as magnetic resonance imaging (MRI) or computerized tomography (CT), can also have difficulty distinguishing small tumors from benign unilateral hypertrophic tonsils [8].

Recent studies have reported an alternative imaging modality using low-frequency transcervical US to improve the diagnostic work-up of patients with OPCs [9,10,11,12,13,14]. Low-frequency transcervical US increases the depth penetration needed to visualize the oropharyngeal surface compared to conventional high-frequency transcervical US but may hinder the detection of smaller OPCs due to reduced image resolution. Fakhry et al. found that transcervical US can increase the tumor detection rate to 70% for patients with unknown primary OPCs, but the tumors still needed to be above 10 mm to be visualized [10]. 

Instead, small-footprint transducers can be used with a transoral technique to obtain direct contact with the mucosa of the palatine and lingual tonsils for improved tumor detection and visualization [15,16]. High-frequency, small-footprint US transducers have recently been developed, resulting in increased superficial soft tissue resolution. We have previously described the use of transoral US for detecting peritonsillar abscesses [17,18]; however, the potential of high-frequency transoral US to diagnose OPCs has not been investigated in any prospective trials. Furthermore, since surgeon-performed neck US is increasingly used worldwide [19,20,21], we believe transoral US may be added to the outpatient clinical work-up.

Our primary objective was to explore the feasibility of surgeon-performed transoral or transcervical US in the outpatient clinic to detect OPCs compared to MRI. Our secondary objective was to compare the T-staging of OPCs between US and MRI.

## 2. Materials and Methods

The study was performed as a prospective diagnostic study at a tertiary cancer center in the Department of Otorhinolaryngology, Head, and Neck Surgery, Copenhagen University Hospital—Rigshospitalet, Denmark. Clinicaltrials.gov registration (NCT05698667), local ethics review board (H-19010203), and data protection agency (P-2019-1) approvals were obtained. Verbal and written consent were obtained for all patients enrolled in the study. The Standards for Reporting Diagnostic Accuracy (STARD) guidelines were used as a framework for reporting the results of this study [22,23].

We recruited patients referred to our head and neck outpatient cancer clinic for diagnostic work-up of OPC from private practicing otorhinolaryngologists, general practitioners, dentists, and other departments participating in the study. Standard clinical work-up included visual inspection, palpation of the oral cavity and oropharynx, digital endoscopy with narrowband imaging of the pharynx and larynx, and neck US [24]. Some patients were included after OPCs were verified by biopsy, if they had a biopsy performed before arriving at our department.

**Figure 1:**
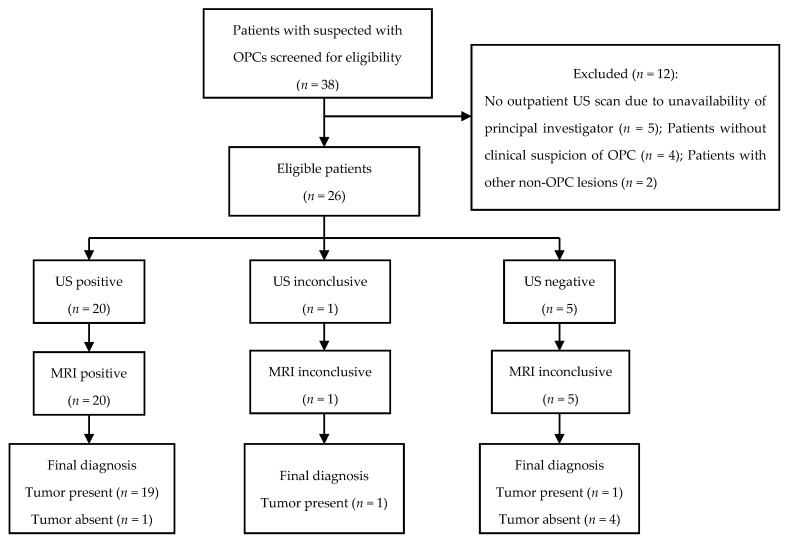
Standards for the Reporting of Diagnostic Accuracy (STARD) flow chart of eligible patients with clinically suspected oropharyngeal cancers (OPC). US: ultrasound; MRI: magnetic resonance imaging.

The inclusion criteria included: patients aged 18+ years with clinically suspected palatine or lingual tonsil cancer (defined as a tumor, asymmetry, ulceration, or unilateral throat or ear pain without any other explanation); patients without available MRIs, due to low tumor suspicion or the unavailability of the principal investigator; and patients with non-oropharyngeal cancers were excluded (Figure 1).

A BK5000 ultrasound machine (BK Medical, GE HealthCare Technologies Inc., Chicago, IL, USA) with the “X18L5s” linear 5–18 MHz hockey-stick transducer was used for transoral US. An “18L5” linear 5–18 MHz or “9C2” curved 2–9 MHz transducer was used for transcervical US. Transoral US was offered to all patients, while transcervical US was offered as an alternative if the clinically suspected location was difficult to reach. The included patients were seated in an examination chair or lying supine on an examination table. A topical anesthetic spray (Xylocain®, Aspen Pharma Trading Limited, Dublin, Ireland) was applied to the oropharynx to reduce the gag reflex (lidocaine 10 mg/dose). The transducer was inserted into a glove containing US gel and placed on the suspected and contralateral palatine tonsils. A sweep was performed from the upper to lower tonsil poles. A power doppler was used to examine differences in vascularity compared to the contralateral palatine tonsil. For transcervical US, the tongue and lingual tonsil were visualized in transverse and sagittal planes in the submental region, according to prior standardized methods provided by Coquia et al. [12]. The patients were asked to extrude their tongue during scanning in order to assess the lingual tonsil dynamically.

The primary outcome was the diagnostic accuracy to detect OPCs and benign tonsil tissue between US and MRI in patients with suspected OPCs. The secondary outcome was to compare the greatest tumor size of the OPC and compare categorical T-staging between US and MRI. 

The following criteria were used to determine tumor detection based on transcervical US protocols: tumors were hypoechoic, had a loss of tonsillar striations, and had altered doppler flow compared to the contralateral tonsil [12]. We pre-specified a “positive” test result as a distinguishable tumor compared to the contralateral side. Asymmetry of the palatine or lingual tonsils where a tumor could not be excluded was registered as “inconclusive”. Palatine or lingual tonsils with symmetrically distributed tonsillar striations were registered as “negative”. The greatest tumor size and T-stage were registered according to the TNM classification system [25]. Clinical information was available to the principal investigator during US scans, including age, sex, smoking habits, clinical features of the oropharynx, and narrowband imaging findings. All US examinations were performed blind to MRI findings. 

Contrast-enhanced MRIs were evaluated by an experienced consultant neuroradiologist blinded to transoral US, transcervical US, clinical examination, and histopathology results. Tumor detection was pre-specified according to the following classifications: a “positive” result was given for clearly visible tumors, an “inconclusive” result for suspiciously asymmetrical tonsil tissue, and a “negative” result if there was no asymmetry or suspect lesions in the oropharynx.

The reference standard for tumor detection was the final histopathology biopsy results from the primary tumor. Results were handled as binary: “tumor” vs. “no tumor”. The HPV status of tumors was also registered, as previously described [4]. Frozen-section biopsy analysis was used in all cases with highly suspected OPCs, which allowed the pathologist to evaluate the specimens within 30 min. After histopathology and cross-sectional imaging results became available, a multidisciplinary conference was held for patients with OPCs, where the final T-stage was determined. This final T-stage was used as a reference standard for comparing the agreement of categorical T-staging with US and MRI. Patients without available histopathology results were excluded.

### Statistical Analysis

For the primary outcome, inconclusive tumor detection results were pooled with positive results because the clinical consequences often led to further invasive procedures or diagnostic tonsillectomy [26]. This dichotomization was necessary to calculate sensitivity, specificity, positive and negative predictive values (PPV, NPV), and overall accuracy. Where applicable, 95% confidence intervals (95% CI) were calculated. The overall accuracy of US and MRI was compared by constructing a 2 × 2 table with the number of correctly and incorrectly identified benign or OPC cases with reference to histopathology. The McNemar’s χ^2^ test for paired data was then used to compare the discordant values in this 2 × 2 table. A *p*-value of <0.05 was considered statistically significant.

For the secondary outcome, we compared the greatest tumor sizes measured with US and MRI in craniocaudal, mediolateral, and anteroposterior diameters. Standard US measurement axes in both transverse and sagittal transducer orientations were pre-determined with author RN to improve the accuracy of correlations with standard MRI measurement axes. Scatter plots and the Pearson’s r correlation coefficient were computed for all three diameters. As a post-hoc test, categorical T-staging was also compared with the following definitions: T1 (tumor < 20 mm), T2 (tumor 20 mm–40 mm), and T3 (tumor > 40 mm or extends to the lingual surface of the epiglottis), and a combined T4a and T4b for p16-negative tumors into a single “T4” stage, which is in line with other studies comparing HPV+ and HPV− tumors [25,27,28,29]. Percentage agreement and Cohen’s Kappa coefficient were computed for categorical T-stage between US and MRI, US and final T-stage, and MRI and final T-stage. Statistical analysis was performed in R software version 4.2.2 (R Core Team, Vienna, Austria) using the “stats”, “epiR”, “irr”, and “ggplot2” packages. 

## 3. Results

### 3.1. Screening and Inclusion

Thirty-eight patients with suspected OPCs were screened for eligibility between 26 October 2021 and 19 April 2022. Twelve patients were excluded because they did not meet the inclusion criteria. Twenty-six patients were enrolled and had a transoral US scan performed in addition to the head and neck MRI (Figure 1). Histopathology results were available for all patients enrolled. Fourteen patients had transoral US performed before the biopsy (54%), while twelve patients were included after OPCs were verified by biopsy (46%).

### 3.2. Reference Standard Diagnosis and T-Stage

Outpatient clinic frozen-section biopsy was sufficient to establish a diagnosis in 17 patients (65%), while nine (35%) required perioperative biopsies or tonsillectomy. The histopathology results revealed twenty-one patients with OPCs (81%) and five patients with benign palatine or lingual tonsils (19%) (see Table 1 for demographics, reported symptoms, and clinical findings). Palatine tonsil cancers were found in 12 of 21 patients with OPCs (46%), lingual tonsil cancers in six patients (23%), and overlapping palatine and lingual tonsil cancers in three patients (12%). Squamous cell carcinoma (SCC) was the most frequent cancer type (*n* = 18, 86%), followed by lymphomas, found in two patients, and one adenoid cystic carcinoma. The final T-stage in the 18 SCCs resulted in two patients with stage T1 (10%), ten patients with T2 (56%), four patients with T3 (22%), and one patient with a T4 tumor (5%). Fourteen SCCs were p16-positive (78%), and four were p16-negative (22%). Of these p16-positive SCCs, 11 tested HPV-positive (79%), one tested HPV-negative, and two were not tested.

### 3.3. Detection of OPCs Compared between US and MRI

**Figure 2:**
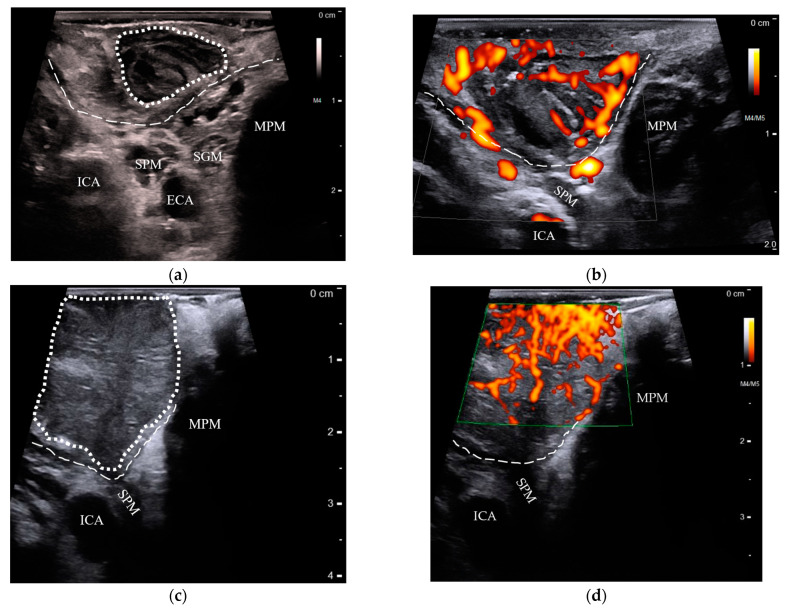
Comparison of transoral US features in benign and cancerous palatine tonsils: (**a**) a normal palatine tonsil (dotted outline) with striated crypts overlying the constrictor muscle (dashed line), lying superficially to the stylopharyngeus muscle (SPM), styloglossus muscle (SGM), medial pterygoid muscle (MPM), internal carotid artery (ICA), and external carotid artery (ECA); (**b**) power doppler shows the palatine tonsils’ blood flow originating from the constrictor muscle and branching outward parallel to the crypts; (**c**) a stage-T2 palatine tonsil HPV+ SCC appears as an enlarged, hypoechoic mass without crypt striations (dotted outline), but respects the boundaries of the constrictor muscle (dashed line); (**d**) with power doppler, the same tumor is seen with random, chaotically increased blood flow. US: ultrasound; HPV+ SCC: human papillomavirus-positive squamous cell carcinoma.

US and MRI test results are shown in Table 1. Transoral US of the palatine tonsils could be performed in all patients with suspected palatine tonsil cancers (*n* = 21), while only one patient could tolerate transoral US of the lingual tonsil. Instead, we performed transcervical US of the oropharynx in the remaining four lingual tonsil cases. Tumors of the palatine tonsils could be distinguished from the contralateral tonsils due to increased size, loss of striated appearance, and chaotic doppler flow (Figure 2, Video S1). 

In the OPC group, US and MRI had one and two inconclusive cases, respectively, which were analyzed as positive tests. In the benign group, MRI had four inconclusive tests, which were analyzed as positive tests. The overall accuracy was 92% (95% CI: 75–99%) for combined US evaluation and 81% (95% CI: 61–93%) for MRI, with no significant difference (*p* = 0.37). Stratifying patients scanned with transoral US and transcervical US alone showed similarly high accuracies of 91% (95% CI: 71–99%) and 88% (95% CI: 47–99%), respectively (Tables S1 and S2). Combined US sensitivity, specificity, PPV, and NPV were 95%, 80%, 95%, and 80%, respectively (Table 2). MRI sensitivity, specificity, PPV, and NPV were 100%, 0%, 81%, and 0%, respectively. 

We performed a post-hoc test comparing the 14 cases included, blinded to histopathology: US sensitivity, specificity, PPV, NPV, and accuracy were 89%, 80%, 89%, 80%, and 86%, respectively. MRI sensitivity, specificity, PPV, NPV, and accuracy were 100%, 0%, 64%, 0%, and 64%, respectively. The overall accuracy was not significantly different (*p* = 0.37) (Table S3).

### 3.4. Comparison of T-Staging between US and MRI

#### 3.4.1. Correlation of the Greatest Tumor Size

**Figure 3:**
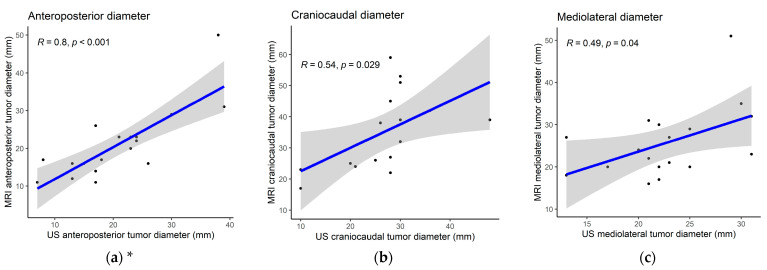
Comparison of primary tumor size between US and MRI in 15 biopsy-verified SCCs (three missing due to an inconclusive US scan): (**a**) anteroposterior tumor diameter; (**b**) craniocaudal tumor diameter; (**c**) mediolateral tumor diameter.* Two cases could not be measured in craniocaudal diameter. US: ultrasound; MRI: magnetic resonance imaging; SCC: squamous cell carcinoma.

**Figure 4:**
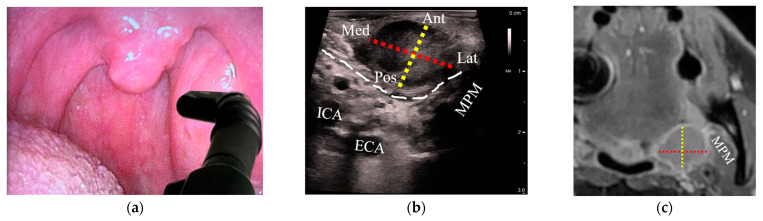

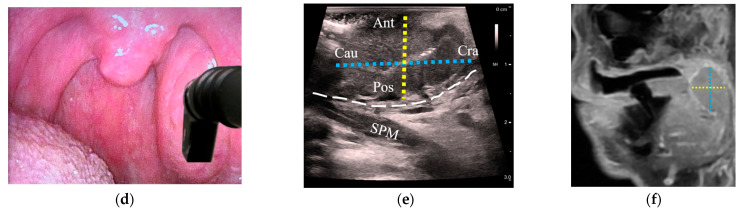
Comparison of tumor diameters between transoral US and corresponding MRI images of a stage-T2 palatine tonsil HPV+ SCC: (**a**) a patient with an asymmetric left palatine tonsil with an inserted hockey-stick ultrasound transducer placed onto the tonsil in a transverse plane; (**b**) transverse US reveals a palatine tonsil tumor. Tumor diameters are represented from medial (Med) to lateral (Lat) with a red dotted line and from anterior (Ant) to posterior (Pos) with a yellow dotted line; (**c**) axial MRI with the corresponding diameters as in (**b**, red and yellow dotted lines); (**d**) transducer oriented in the sagittal plane; (**e**) sagittal US with the cranial (Cra) to caudal (Cau) diameter added (blue dotted line) along with Ant-Pos (yellow dotted line); (**f**) sagittal MRI with the corresponding diameters as in (**e**, blue and yellow dotted lines). US: ultrasound; MRI: magnetic resonance imaging; HPV+ SCC: human papillomavirus-positive squamous cell carcinoma; ICA: internal carotid artery; ECA: external carotid artery; MPM: medial pterygoid muscle; SPM: stylopharyngeus muscle; white dashed line: constrictor muscle; Ant: anterior; Pos: posterior; Med: medial; Lat: lateral; Cra: cranial; Cau: caudal.

Tumor size was estimated with both US and MRI in 18/21 patients with OPCs (86%); three patients whose tumors could not be delineated on US were excluded from analysis. In all cases, outpatient US was performed a median of 5 days after MRI (interquartile range: −2 to 10 days). A high correlation between US and MRI was observed when comparing the tumor diameter along the US depth axis, which corresponded to the anteroposterior diameter (R = 0.80, Figure 3a). The lateral US axes corresponding to craniocaudal (R = 0.54) and mediolateral diameters (R = 0.49) demonstrated moderate correlation with MRI (Figure 3b,c). Figure 4 illustrates transoral transducer orientations and the corresponding US axes compared to MRI.

#### 3.4.2. Agreement of Categorical T-Stage

When comparing US and MRI to the final T-stage, an agreement of 53% and 61% and a weighted kappa of 0.25 (fair agreement) and 0.43 (moderate agreement) were found, respectively (Table S4).

## 4. Discussion

To the best of our knowledge, this is the first prospective clinical trial to explore the feasibility and accuracy of outpatient-performed transoral US for the diagnostic work-up of oropharyngeal cancers. We found that transoral US is feasible to perform as an extension of the clinical examination (85% tolerated the exam) with a diagnostic accuracy of 92%, which was comparable to MRI with 81% (*p* = 0.37). We also found that tumor diameters corresponding to the depth axis on US correlated well with MRI. 

Our primary objective was to explore the detection of OPCs with oropharyngeal US compared to MRI, and we found sensitivities of 95% and 100%, respectively. Since we included all patients with suspected OPCs, our study sample included two palatine tonsil lymphomas and one lingual tonsil adenoid cystic carcinoma. Transoral US may distinguish a tonsil lymphoma from the contralateral tonsil, but distinguishing lymphomas from unilateral chronic tonsillitis may be difficult; therefore, a combined clinical evaluation, transoral US, and large biopsies or tonsillectomy will secure a diagnosis. Due to the increased detail of the palatine tonsils with transoral US, we found a promisingly high specificity with transoral US compared to MRI for evaluating patients with benign unilateral tonsil hypertrophy; therefore, transoral US could possibly prevent unnecessary diagnostic tonsillectomies. MRI could not distinguish benign cases from OPCs, which resulted in 0% specificity; however, the sample size of benign cases was small, and we cannot conclude whether there is a significant difference in specificity from the current data.

Prior studies have reported similarly high unblinded sensitivities of 90–100% with transcervical US for detection of OPCs [9,13,14]. In our study, due to the prospective inclusion of 14 (54%) patients blinded to histopathology, we were also able to report a specificity for oropharyngeal US. We also found transoral US feasible in all cases with palatine tonsil OPCs, but, due to gag reflex, we mostly performed transcervical US for patients with suspected lingual tonsil OPCs. The lingual tonsil is a very gag-sensitive area and requires greater local anesthesia, smaller US equipment, and a cooperative patient. Thus, transcervical US may be more feasible for lingual tonsil OPCs. 

The secondary objective was to explore T-staging with US. We found a high correlation (R = 0.80, *p* < 0.01) in anteroposterior tumor diameters between US and MRI. A previous transcervical US study reported similar results when comparing US to standard imaging (MRI and CT) for OPC tumors (*n* = 35), with the highest correlation in the anteroposterior diameter (R = 0.71) [13]. They also reported that the dynamic off-axis views from US are difficult to match up perfectly with static cross-sectional imaging. Like this previous study, we found moderate correlations in craniocaudal and mediolateral diameters. In our study, this was due to the limited lateral field of view of the hockey-stick transducer. As a result, tumors longer or wider than 30 mm were difficult to estimate properly. Controversially, a previous retrospective study using a wider-field-of-view transoral US with low-frequency transducers found similar results, with MRI proving to be superior for T-staging larger tumors [15]. To explore this further, future studies are required where a combined transoral and transcervical ultrasounds of the oropharynx is performed in all cases.

Due to the limited field of view of small transoral US transducers, large T3 tumors were difficult to measure. In contrast, MRI is not hindered by larger tumors. High-frequency transoral US could instead add valuable information regarding tumor depth of invasion, similar to studies from intraoral US of oral tongue squamous cell carcinomas [30,31,32]. Further, it is well known that MRIs can overestimate tumor sizes due to an edema surrounding the tumor or due to biopsy trauma [33]. A potential T1–T2 tumor could therefore be wrongly excluded from surgical treatment due to inadequate delineation from deeper structures on MRI. As a result, tumor borders may be more clearly visualized with transoral US since this examination can be performed prior to biopsy.

The clinical implications of adding transoral US during the objective evaluation of patients with OPCs include the potential to distinguish between benign hypertrophic tonsils and OPCs. Thus, excessive MRIs or tonsillectomies in patients with benign unilateral palatine tonsil hypertrophy could be avoided. Our study does not include cases of inflammatory tonsils, which have altered ultrasonographic features that can mislead clinicians. It is therefore important to include clinical findings when evaluating transoral US findings. Furthermore, transoral US also allows for evaluating tumor involvement in the parapharyngeal space, including the deep vasculature, which is important for surgical planning [34,35]. With proper transoral US training, head and neck cancer clinics may improve their clinical certainty factor for detection and T-staging of OPC patients. As surgeon-performed transcervical US is gaining popularity, the addition of a hockey-stick transducer is a relatively low-cost investment for point-of-care OPC detection and T-staging. Fast and accurate T-staging allows the clinician to better plan the treatment course for the patient, whether it be surgical or radiotherapy. 

Our study has some limitations. Firstly, we included 12 patients with biopsy-verified OPCs, causing a risk of bias in US sensitivity. To reduce this risk, we predetermined that positive test results required a clearly visible tumor on the US. We controlled this by performing a post-hoc test on patients included before histopathology-verified diagnosis, resulting in 86% accuracy compared to 92% for all cases. Regarding T-staging, our hypothesis was that high-frequency transoral US would improve the visualization of smaller OPCs. The small sample size was one limitation of this study. Due to this limitation, we cannot generalize our results broadly, especially considering the low number of T1 and T4 cases. This also limits the statistical power of our study. We intended to explore the feasibility of outpatient transoral US, which showed promising results. A larger multicenter study with prospective inclusion of patients blinded to histopathology and MRI would address the true diagnostic accuracy of US and MRI while also addressing a learning curve for transoral US. To understand which imaging modality most accurately T-stages OPCs, a study comparing tumor sizes and categorical T-staging in surgically resected specimens between US and MRI, using pathologic tumor size and T-stage as references, is required. 

## 5. Conclusions

In this small study, we found that outpatient-performed transoral US was a feasible and accurate diagnostic tool comparable to MRI for detecting tumors of the palatine tonsils; however, transcervical US may be more practical for imaging the lingual tonsils due to the gag reflex that can occur with transoral US. Clinical T-staging with transoral US shows promise, but larger tumors can be difficult to accurately measure.

## Figures and Tables

**Table 1:** Demographics and clinical characteristics of the patients included in the final analysis.

Variables	All Patients (*n* = 26)	OPC (*n* = 21)	Benign (*n* = 5)
	No. (%)	No. (%)	No. (%)
**Sex**			
*Female*	14 (54)	10 (48)	4 (80)
*Male*	12 (46)	11 (52)	1 (20)
**Age, median years [IQR]**	63 [56.5; 71]	63 [59; 72]	58 [55; 61]
**Smoker (>10 pack years)**	17 (65)	14 (67)	3 (60)
**Objective findings**			
*Visible tumor*	14 (54)	14 (67)	0 (0)
*Submucosal tumor*	6 (23)	5 (24)	1 (20)
*Unilateral hypertrophy or pain*	6 (23)	2 (10)	4 (80)
**Clinically suspected tumor site**			
*Palatine tonsil*	21 (81)	17 (81)	4 (80)
*Lingual tonsil*	5 (19)	4 (19)	1 (20)
**Primary US method used**			
*Transoral*	22 (85)	18 (86)	4 (80)
*Transcervical*	4 (15)	3 (14)	1 (20)
**US test results**			
*Positive*	20 (77)	19 (90)	1 (20)
*Inconclusive*	1 (4)	1 (5)	0 (0)
*Negative*	5 (19)	1 (5)	4 (80)
**MRI test results**			
*Positive*	21 (81)	19 (90)	1 (20)
*Inconclusive*	5 (19)	2 (10)	4 (80)
*Negative*	0 (0)	0 (0)	0 (0)

OPC: oropharyngeal cancer; IQR: interquartile range; US = ultrasound.

**Table 2:** Overview of oropharyngeal tumor detection positive, negative, and inconclusive results from MRI, transoral US, transcervical US, and combined US evaluation.

Diagnostic Test	Se (95% CI)	Sp (95% CI)	PPV (95% CI)	NPV (95% CI)	Acc (95% CI)
MRI	100% (84–100%)	0% (NA)	81% (61–93%)	0% (NA)	81% (61–93%)
Overall US	95% (76–99%)	80% (28–99%)	95% (76–99%)	80% (28–99%)	92% (75–99%)
Transoral US	94% (73–99%)	75% (19–99%)	94% (73–99%)	75% (19–99%)	91% (71–99%)
Transcervical US	86% (42–99%)	100% (NA)	100% (54–100%)	50% (13–99%)	88% (47–99%)

Se: sensitivity; 95% CI: 95% confidence interval; Sp: specificity; PPV: positive predictive value; NPV: negative predictive value; Acc: overall accuracy; MRI: magnetic resonance imaging; US: ultrasound; NA: not applicable.

## Data Availability

The data are contained within the article or supplementary material. The data presented in this study are available within the supplementary material in Table S1.

## Supplementary Materials

The following supporting information can be downloaded at: https://www.mdpi.com/article/10.3390/cancers15215292/s1, Table S1: A 2 × 2 table presenting overall accuracy of US and MRI; Table S2: Participant data; Table S3: Tumor detection of US and MRI in 14 cases blinded to histopathology; Table S4: T-stage categorical values for US, MRI, and final diagnosis; Video S1: Transoral ultrasound of a benign and cancerous palatine tonsil.
