# Supplementary material for: Transoral Ultrasound in the Outpatient Clinic for the Diagnostic Work-Up of Oropharyngeal Cancer: A Feasibility Study
Source: Cancers (Basel). 2023 Nov 4;15(21):5292. doi: 10.3390/cancers15215292 (PMC10649062; doi:10.3390/cancers15215292)
Supplement: Supplementary file 1 [file cancers-15-05292-s001.zip › cancers-2674062-supplementary.zip!/cancers-2674062-supplementary.pdf]

Supplementary Materials: Transoral Ultrasound in the Outpatient Clinic for the Diagnostic Work-Up of Oropharyngeal Cancer: A Feasibility Study

Martin Garset-Zamani, Rikke Norling, Christoffer Holst Hahn, Tina Klitmøller Agander, Christian von Buchwald and Tobias Todsen

Table S1. 2 × 2 table overall accuracy of US and MRI.

	MRI		Total
US	Correct	Incorrect	
Correct	20	4	24
Incorrect	1	1	2
Total	21	5	26

McNemar's test with continuity correction: $(|4-1|-1)^2/(4+1) = 0.8$, $p=0.37$.

Table S2. Participant data.

Age, Sex	Clinic suspected location	US method / blinded to histopathology	US result / location	MRI result / location	MRI / US primary tumor size (mm)	MRI / US T-stage	Final diagnosis / location	Final T-stage
**55, F**	LIN	TC / Yes	- / NA	? / LIN	NA	NA	No tumor	NA
**63, F**	PAL	TO / Yes	- / NA	? / PAL	NA	NA	No tumor	NA
**55, F**	PAL	TO / Yes	- /NA	? / PAL	NA	NA	No tumor	NA
**81, F**	PAL	TO / Yes	+ / PAL	+ / PAL	45 / 28*	3 / 2	LYM / PAL	NA
**63, F**	PAL	TO / No	+ / LIN	+ / PAL	35 / NA	2 / NA	SCC / LIN	2
**71, F**	PAL	TO / No	+ /PAL	+ / PAL	19 / 21	1 / 2	SCC / PAL	2
**75, F**	PAL	TO / No	+ / PAL	+ / PAL	51 / 30	3 / 2	SCC / PAL	3
**71, M**	LIN	Both / No	+ / LIN	+ / LIN	23 / 25	2 / 2	SCC / LIN	2
**67, M**	PAL	TO / Yes	+ / PAL	+ / PAL	53 / 30*	3 / 2	SCC / PAL	3
**63, F**	PAL	TO / No	+ / PAL	+ / PAL	26 / 17	2 / 1	SCC / PAL	2
**61, F**	PAL	TO / Yes	+ / PAL	+ / PAL	30 / 25*	2 / 2	No tumor	NA
**50, M**	PAL	Both / Yes	+ / PAL	+ / LIN	35 / 30*	4 / 2	SCC / Uns	3
**72, M**	PAL	Both / No	+ / LIN	+ / Uns	59 / 38	4 / 4	SCC / Uns	42
**52, M**	LIN	TC / No	+ / LIN	+ / LIN	24 / 21	3 / 2	SCC / LIN	1
**58, M**	PAL	TO / Yes	- / NA	? / PAL	NA	NA	No tumor	NA
**56, M**	PAL	Both / Yes	- / NA	? / PAL	34 / NA	2 / NA	SCC / LIN	1
**68, F**	PAL	TO / Yes	? / PAL	? / PAL	28 / NA	2 / NA	SCC / PAL	2
**49, M**	PAL	TO / Yes	+ / PAL	+ / PAL	32 / 30*	2 / 2	SCC / PAL	2
**63, F**	PAL	TO / Yes	+ / PAL	+ / PAL	25 / 20	2 / 1	LYM / PAL	NA
**61, F**	PAL	TO / Yes	+ / PAL	+ / PAL	39 / 30*	2 / 2	SCC / PAL	2
**52, F**	PAL	TO / No	+ / PAL	+ / PAL	27 / 13	2 / 1	SCC / PAL	3
**75, M**	PAL	TO / No	+ / PAL	+ / PAL	38 / 26*	2 / 2	SCC / PAL	2
**72, F**	LIN	TC / No	+ / LIN	+ / LIN	22 / 28	2 / 2	SCC / LIN	1
**63, M**	PAL	TO / Yes	+ / PAL	+ / PAL	27 / 28	2 / 2	SCC / PAL	2
**59, M**	PAL	TO / No	+ / PAL	+ / PAL	42 / 36	3 / 2	SCC / PAL	2
**73, M**	LIN	TC / No	+ / LIN	+ / LIN	39 / 48	4 / 4	AdCC / LIN	3

Case overview with clinical, US, and MRI. Suspected tumor locations are noted, along with the primary tumor size in millimeters. **These tumors were larger than the transoral US probe and thus inaccurately measured. US = ultrasound, MRI = magnetic resonance imaging, F = female, M = male, NA = not available, TC = transcervical, TO = transoral, LIN = lingual tonsil, PAL = palatine tonsil, Uns = unspecified*

or overlapping oropharyngeal subsites, + = positive test, - = negative test, ? = inconclusive, LYM = lymphoma, SCC = squamous cell carcinoma, AdCC = adenoid cystic carcinoma.

Table S3. Tumor detection of US and MRI in 14 cases blinded to histopathology.

	TP	TN	FP	FN	Se (95% CI)	Sp (95% CI)	PPV (95% CI)	NPV (95% CI)	Acc (95% CI)
**MRI**	9	0	5	0	100% (66-100%)	0% (NA)	64% (35-87%)	0% (NA)	64% (35-87%)
**US**	8	4	1	1	89% (52-99%)	80% (28-99%)	89% (52-99%)	80% (28-99%)	86% (57-98%)

TP = true positive, TN = true negative, FP = false positive, FN = false negative, Se = sensitivity, CI = confidence interval, Sp = specificity, PPV = positive predictive value, NPV = negative predictive value, Acc = overall accuracy, MRI = magnetic resonance imaging, US = ultrasound, NA = not applicable.

Table S4. T-stage categorical values for US, MRI, and final diagnosis.

US vs. Final T-stage	US: T1	US: T2	US: T3	US: T4
Final: T1	0	2	0	0
Final: T2	1	7	0	0
Final: T3	1	3	0	0
Final: T4	0	0	0	1
MRI vs. Final T-stage	MRI: T1	MRI: T2	MRI: T3	MRI: T4
Final: T1	0	2	1	0
Final: T2	1	8	1	0
Final: T3	0	1	2	1
Final: T4	0	0	0	1

Blind evaluated T-stage agreement between US, MRI, and final consensus T-stage for 18 patients with oropharyngeal squamous cell carcinoma.

Video S1: Transoral ultrasound of a benign and cancerous palatine tonsil.

Video S1 is provided separately, attached as an .mp4 file.
